# Management Issues for Adolescents with Cystic Fibrosis

**DOI:** 10.1155/2012/134132

**Published:** 2012-09-06

**Authors:** Adelaide Lindsay Withers

**Affiliations:** Department of Respiratory Medicine, Princess Margaret Children's Hospital, Level 3, Harry Boan Building, Roberts Road, Subiaco, WA 6009, Australia

## Abstract

The healthy adolescent will encounter major changes in biological and psychosocial domains. The adolescent period can be greatly affected by a chronic illness. Cystic fibrosis is a terminal illness that can significantly affect an adolescent's biological, mental and psychosocial health. This paper discusses general issues to consider when managing an adolescent with a chronic medical condition, and specifically how cystic fibrosis may impact upon puberty, body image, risk-taking behaviours, mental health, independence, nonadherence, reproductive health, transition, lung transplantation, and end of life care.

## 1. Introduction

Adolescence can be a tumultuous and demanding time for the teenager and their family. The adolescent will undergo great changes in all domains of life, including physical, biological, cognitive, developmental, and social, as well as the transition from being a dependent child to an independent adult [1]. The additional complexity of a chronic illness can greatly complicate adolescence, impair the achievement of the normal goals of adolescence and requires special consideration when providing medical care. Adolescents are likely to engage in risk-taking behaviours that can have far-reaching health implications if a chronic illness is thrown into the mix. Chronic illness may make risky behaviours more likely to occur in a bid to gain acceptance from peers or feel more “normal,” especially given the need of adolescents to conform and “fit in.” Healthy adolescents often have concerns about puberty and body image that may be magnified by a chronic illness. This is especially common with an illness like cystic fibrosis (hereafter CF), which can produce very visible symptoms by affecting pubertal status and stature [2, 3]. An additional concern for adolescents with a chronic illness is how their disease will affect their future—they may wonder if they will be able to finish school, have a job, have sexual relationships, or start a family [2–5]. Unfortunately, adolescents with a chronic illness have been reported to have fewer friends, be less likely to develop relationships with a person of the opposite sex [5–8], and may require a great deal of reassurance and sensitive exploration of these issues. 

CF is the most common inherited, autosomal recessive disorder in Caucasians, with a prevalence of approximately 1 in 2500 live births [9]. The underlying abnormality lies in the chloride ion channel encoded by the cystic fibrosis transmembrane conductance (CFTR) gene [10]. CF affects most organ systems, with respiratory and gastrointestinal being the most severely affected. Although CF is still considered a terminal disease, life expectancy has dramatically increased from 14 years of age (1969) to 32 years of age (2000) [9]. Children with CF born after the year 2000 can be expected to survive well into their 50's [11]. The usual cause of death is respiratory failure. As the life expectancy increases in CF, patients are providing challenges to paediatric teams who are not used to managing medical problems unique to adolescents and young adults [12]. Although CF care that is adolescent appropriate should be standard, patients unfortunately have unmet needs such as reproductive health, sexuality, pregnancy, and mental health. Although these issues are commonly encountered in healthy adolescents, CF itself can greatly complicate management of these already difficult areas. In addition, attention to the psychosocial wellbeing of adolescents with CF is of paramount importance and, sadly, often ignored. 

Managing adolescents with CF can be a difficult and frustrating task, but the ability to work effectively with an adolescent is highly rewarding. The primary relationship shifts from being between the physician and the parents to one between the physician and the adolescent, which makes seeing the young person on their own essential [1]. It is a privilege to observe, accompany, and encourage the young person on their journey to becoming an independent adult who can effectively manage their medical condition. This paper outlines some of the general aspects of providing appropriate medical care to adolescents, with an emphasis on the effects of CF on the adolescent years and some of the issues that arise. Topics covered include general considerations for adolescents, puberty and body image, drug use, mental health, developing independence, nonadherence, reproductive health, transition, lung transplantation, and end of life care. 

## 2. General Considerations

Ideally, some of the consultation should occur with the adolescent on their own. This can be challenging for physicians, especially when they have been managing a patient since infancy and are used to conducting the majority of the consultation via the parents. Adolescents and young adults with CF have expressed that they should be seen on their own (for at least part of the consultation) between the ages of 13 and 16 years [13]. Importantly, patients indicated their wish to discuss adolescent issues with their doctor in private, but not all were given that opportunity [13]. It is useful to introduce the idea of seeing adolescents on their own around the ages of 12-13 years. Asking the adolescent for permission is useful, and gentle explanation to the parents and teenager that this is standard practice can often allay anxieties about “secrets” or collusion. Seeing the adolescent on their own can be useful after starting the appointment with the parents to address medical concerns, while directing questions to the adolescent and encouraging them to answer questions for themselves.

Once the adolescent is seen alone, it is of the utmost importance that they understand the concept of confidentiality. Explain to them that it is important to see them alone as there may be issues they prefer not to discuss with their parents present, and this is a normal part of developing independence and responsibility for their health. Confidentiality must be ensured, and the teenager must understand that the only time confidentiality can be broken is if someone is hurting them, they are hurting themselves, or they might hurt someone else. It is also useful to discuss that sometimes information might need to be shared with other team members, but only with the adolescent's permission. Discussing confidentiality establishes a sense of trust in the health professional and may allow for a more open and honest discussion. 

The issue of providing consent can be difficult and depends on the nature of the proposed treatment and the age of the adolescent. Under common law in Australia, adolescents under the age of 18 may be able to provide informed consent if the adolescent is deemed by the treating doctor to be competent to understand the nature of the treatment, all consequences of accepting or rejecting treatment, and alternative options to treatment. If these criteria are satisfied, the adolescent is considered a “mature minor” and can consent to treatment without parental consent. It is imperative to formally document this process, encourage the adolescent to inform their parent, and explore what may happen if their parent finds out. Obviously, the nature of the treatment and gravity of consequences must be considered (e.g., consenting to take antibiotics for sinusitis is very different to consenting to a termination of pregnancy). Careful consideration must be given to child protection issues and whether a mandatory report is required (e.g., requesting contraception in sexual abuse cases). For this reason, it is essential to discuss confidentiality as outlined above and make the exceptions clear so that in situations where confidentiality must be breached for child protection or legal reasons the adolescent does not feel betrayed and you have not made promises that you cannot keep. Generally a parent will sign a consent form for surgical procedures until the age of 18 although an adolescent should be encouraged to give informed consent and sign as well. In difficult cases it is wise to seek legal advice. 

When seeing an adolescent on their own, it is useful to remove the focus from medical issues and perform an adolescent health screen. Many use the acronym HEARDDSS as a reminder: enquire about the home environment, education, activities they like to engage in, relationships (with friends, family, and opposite/same sex), drug use (smoking, alcohol, and illicit drugs), depression, sexual activity (contraception, sexually transmitted diseases), and suicide risk/risk-taking behaviours. It is useful to preface this by discussing confidentiality and normalising the questioning process “We ask everyone your age these questions.” You are more likely to get an honest response by normalising the scenario “Lots of people your age experiment with drinking alcohol—do any of your friends drink at parties? How about you?”

## 3. Puberty and Body Image

Many CF health-related problems become more “visible” during the adolescent years [11]. Unfortunately, adolescence is also a time when teenagers are acutely focused on their appearance, body image, conforming, and comparing themselves to their peers. Self-worth and body image is strongly tied to how well they conform. Any differences from peers can be particularly distressing to an adolescent and could contribute to the development of anxiety and depression. CF affects almost all body systems and produces a wide range of symptoms that patients may find very embarrassing and distressing [14, 15]. An Australian survey found that symptoms seen commonly in CF (coughing, flatulence, steatorrhoea, discoloured teeth from antibiotics, and digital clubbing) were extremely distressing and isolating for adolescents, especially those that cause visible difference to their peers (pubertal delay, short stature, and low body mass) [15]. Patients with CF began to realise that they were different from healthy children even prior to the age of three years [15]. Gastrointestinal symptoms had forced many adolescents to miss out on school camps, outings, and social activities, solely due to embarrassment [15]. Teenagers often complained that they could only fit into children's clothing and shoes, and fashionable clothing was not available in their size [15]. Pubertal delay is common in CF even when nutritional status is normal, with an average of 2-year delay in females and 1.5 years in males [16]. Lack of pubertal delay was a major issue for over 60% of adolescent girls, and lack of breast development had been the trigger for a suicide attempt in an 18-year-old female [15]. Other studies have reported the negative association between short stature, delayed puberty, and body image [17, 18]. Having to take pancreatic enzymes in front of peers or doing chest physiotherapy again mark the adolescent as different, and it has been shown that peers often remark on these differences in a negative fashion [15]. Adolescents with CF are frequently concerned that they will not be attractive to the opposite sex [14]. Delayed puberty and other features of CF that mark the patient as “different” must be discussed in a gentle and sensitive manner. Using an older patient as role model, reassuring that a normal puberty will occur in the near future or offering to allow friends to attend appointments to learn more about CF may be useful in supporting the young person.

## 4. Drug Use: Alcohol, Tobacco, and Other Drugs

Adolescence is a time for experimentation and risk taking, so it is not surprising that teens often try smoking, drinking alcohol, and drugs. There are conflicting reports as to whether adolescents with a chronic medical condition are more or less likely to smoke, abuse drugs and drink alcohol [19]. Some studies have found equal or greater risk-taking behaviours in chronic illness (alcohol and drug use sexual activity) [19], whereas other studies have found that adolescents with a chronic illness are less likely to engage in these behaviours [1]. Research has found that adolescents with CF may be less likely to smoke cigarettes than healthy peers [1, 20]. A self-report survey of American adolescents with CF found that they reported less alcohol, tobacco, and drug use than age-matched peers and if they did partake in these behaviours, they often began at an older age [20]. Regardless of these findings, adolescents in general and with CF are likely to experiment with smoking, drinking alcohol, and using drugs, especially if their friends engage in these behaviours. Risk-taking behaviours may be tried as a way to conform, minimise differences from peers, and try to forget about the burden of CF. Many teenagers with CF express that they will “have fun and enjoy” themselves given the terminal nature of CF, which can be a particularly difficult issue to discuss. Unfortunately, explaining how smoking will cause a rapid decline in lung function and lessen life expectancy is rarely useful, as most adolescents do not have the cognitive capacity to understand future consequences as a direct result of their actions. Instead, sensitive discussion using immediate consequences can be a useful strategy; for example, discussing bad breath and unattractive nicotine staining can be useful deterrents to smoking. Regardless of any discouragement, many adolescents will experiment and if they choose to do so, encouraging harm minimisation is essential. Alcohol use (specifically binge drinking) is discouraged, as pancreatitis can result. An additional danger is alcohol-induced hypoglycaemia in CF-related diabetes. If an adolescent chooses to drink alcohol, ask them not to binge drink and make sure they have a sober friend to watch over them. A means of transport to an emergency department if they become unwell is essential, and they must not get in a vehicle with someone who has been drinking. If they develop abdominal pain or begin vomiting, they should present to an emergency department. They need to be aware of the risk of hypoglycaemia and how to prevent and manage this effectively.

## 5. Mental Health: Depression, Anxiety, and ****Suicide

Depression and anxiety have a negative impact on health outcomes, adherence to treatment, health-related quality of life, and risk-taking behaviours. Unfortunately, mental health in CF is often not a focus during adolescence, as new medical problems such as declining pulmonary function, CF-related diabetes, pubertal delay, and osteopaenia may require much attention. As adolescence itself is a time of great change in physical, mental, cognitive, emotional, and social domains, the turmoil of adolescence can render the teen vulnerable to developing a mental health disorder. It is well known that depression and suicidal ideation are common in adolescents; the estimated prevalence of major depression in children and adolescents is between 3 and 5% [21, 22]. It is not surprising that having any kind of chronic illness (particularly one that is life limiting) increases the risk of depression in all age groups [21–23]. The increased risk of psychiatric disorders in chronic illness in children can be up to three times higher than the general population [24]. Although there is much literature examining the psychosocial impact of CF on families (particularly maternal mental health), there is little published research regarding the mental health impact of CF in children and adolescents. 

In general, research has found that psychosocial functioning in CF is similar to those without CF, but mental health problems like depression and anxiety are reasonably common in CF adolescents [12, 15, 21, 25, 26]. Studies that reported a much higher rate of psychopathology in adolescents with CF compared to healthy peers tend to be older studies, when prognosis was far worse and CF was associated with a poor quality of life [26–30]. However, poor quality of life (especially in the physical domain) occurs when CF is severe, and corresponding psychiatric problems commonly occur as the disease progresses [12, 31]. Perhaps the general high quality of life reported by adolescent patients with CF in more recent times accounts for the lower levels of psychiatric disorders [27]. An Australian study of 52 adolescents with CF found that self-reported quality of life was typically high and that only 12.5% of those aged over 13 years reported symptoms of psychopathology (anxiety and obsessive compulsive symptoms) [27]. It is important to note that this rate is lower than the reported rate of psychopathology in healthy adolescents (15–20%) of the same age. The individuals with symptoms of psychopathology had significant risk factors (poor quality of life, late diagnosis, poor family support, and lack of hope for the future) [27]. Families who are nonsupportive have been shown to be the strongest predictor of poor psychological adjustment in patients with CF [27]. Other reports of depression in adolescents with CF tend to be case reports or indirect reports by other family members [15].

Prevalence of depression in adult CF patients has been shown to be higher than the general population, with estimates that range from 29 to 46% [12, 32–35]. One study of adults with CF reported that 20% had mild-to-moderate depression, and 10.5% had moderate-to-severe depression [33]. Pearson et al. [34] reported a prevalence of 22.2% for anxiety and 42.4% for depression in adults with CF. Adult males with CF have been reported to have a higher incidence of psychiatric disorders than females [29]. 

Many studies highlight the fact that not only is depression common in adolescents with CF but has significant negative effects on quality of life, adherence to treatment, and family functioning (which is vital to effective care for an adolescent with CF) [21, 27, 33, 36, 37]. For this reason, Quittner et al. [21] recommend that screening for depression is routine in adolescents with CF and that validated screening tools are available for this purpose. Identification of depression and adequate treatment can have a positive impact not only on a patient's health, but their quality of life and compliance with therapy. 

Chronic illness of any sort in adolescents is known to be associated with an increased risk of suicide attempts, especially in females [38, 39]. A survey of American teenagers with CF found that they were not more likely to have suicidal thoughts than healthy age-matched peers [20]. Prevalence of completed suicide or attempts is not commonly reported for adolescents with CF. A suicide attempt in an 18-year-old female with CF (prompted because of lack of pubertal development) was reported by Allan et al. [15], and most other case reports of suicide attempts in adolescents with CF (in some cases by overdose of medications) come from the 1960's and 1970's when prognosis and quality of life were far poorer [40]. Cases of death due to suicide in CF in young adults are reported in several mortality audits from all over the world, with very small numbers (*n* < 2 or approximately 1% of the cohorts) [41–47]. Reports from the CF Centres Registry in USA (estimated to capture 85% of all patients) between 1988 and 1992 estimated death from suicide or trauma in 1.8% of male deaths and 1% of female deaths; however, trauma and suicide were not separated in this report [48].

It is not surprising that when health-related quality of life is impaired, depression, suicide attempts, and anxiety are common. The events that render an individual with CF more prone to developing a mental health disorder include transition, deterioration in health/quality of life, a new bacteria cultured from sputum, considering pregnancy, transplantation, and during adolescence [49]. CF treatment can be a significant burden, and adding the “general emotional turbulence of adolescence” may contribute to an increased risk of mental health problems in adolescents with CF [12]. The physician must be vigilant in screening, detecting, and treating any mental health disorder, as it may significantly impact upon quality of life and treatment. 

## 6. Becoming Independent and Functioning as an Adult

Although older adolescents with CF are on the brink of having to function in an independent manner, the demands of the illness may hamper the process [50]. Parents have the difficult and unenviable task of continuing to oversee medical treatment, while allowing the teenager to develop their own independence. The quest for independence can be particularly difficult, as the very nature of CF enforces dependency [40] and encourages an “infantilised” self-image [51]. This view is often consciously or unconsciously reinforced by a paediatric health care system. This may impede transition and the adolescent's ability to become independent. Adolescents with CF may fear independence, as juggling employment, tertiary education, and starting a family with their significant health care needs is a daunting task. Young adults with CF are less likely to be employed [26], may fear that disclosing their medical condition will cause potential employers to refuse them [52], and express concern regarding limited opportunities for employment [15]. These problems are compounded by the financial strain many families living with CF face. This is distressing for an adolescent who may blame themselves for causing financial hardship and wonder how they can provide for themselves if they cannot be employed [15]. Some groups have reported males with CF to have higher rates of psychiatric disorders, and this may be related to the inability to be a provider for themselves and others [29, 53]. It has been shown that young adults with CF are more likely to live with their parents [53, 54]. Fear of independence may make transition very difficult, and adolescents may mistakenly believe that nonadherence to treatment may delay transition (unfortunately, this may have the opposite effect, and physicians may bring transition forward out of sheer frustration). Gentle and sensitive exploration with the adolescent to tease out fears they may have, and identification of barriers to developing independence is required. Acknowledgment and affirmation “I can see how this would be scary for you” and identifying supports to assist the transition towards independence are essential.

## 7. Nonadherence to Therapy

Mean adherence rate to long-term medications in adolescents with a chronic illness has been reported as between 33 and 94%, with specific rates of 41–61% reported in CF [55]. Many adolescents become nonadherent to therapy, which can be extremely problematic and even viewed as a form of self-harm. An Australian CF study reported that almost 50% of patients had been nonadherent, usually during the adolescent period [15]. Reported barriers to treatment adherence in adolescents with CF include less parental supervision, a wish to conform to peers, not feeling benefits from treatment, forgetting, rebellion, feelings of futility, disagreeing with the physician, and difficulties with time management [55, 56]. Adolescents may have new conditions such as CF-related diabetes that increase treatment burden even further, compounding the issue. In addition, they often have very busy social lives and may not take kindly to the imposition that treatment may bring.

The best strategy to tackle nonadherence is to explore (in a sensitive, nonjudgemental fashion) the actual reasons for non-adherence and identify the barriers to change. Explain that some degree of non-adherence and forgetting is normal [57]. Demonstrate to the adolescent that you are genuinely interested in why they do not adhere, as they are more likely to honestly disclose information if they feel that you are working with them and not acting as the “medication police.” Involving the adolescent in problem solving, setting realistic goals, and allowing some compromise increases the chance of adherence. Acknowledging the high treatment burden and the unfairness of the situation can engender a sense of “solving this problem together.” Sometimes the issue may be as simple and easily addressed as not being able to swallow a particular tablet or wanting to take medication in private while at school. Other reasons may be more complex, such as frustration about the futility of treatment in an ultimately terminal illness, which can be particularly difficult to discuss. As outlined above, drawing attention to the immediate consequences of not taking medication can be useful, as emphasis on immediate quality of life is more relevant to the adolescent. Methods to improve treatment adherence include having a definite end date to review treatment (making treatment more finite), assisting patients with scheduling and time management, allowing some degree of choice, setting realistic goals, negotiating treatments based on amount of time required, and motivational interviewing [11, 55]. When faced with non-adherence, the ability to listen, compromise, and negotiate with an adolescent is invaluable. 

## 8. Reproductive Health

Despite the hopes and beliefs of many physicians, sexual activity can, and does, occur in adolescents with CF [58, 59]. Although a survey of American teens with CF found that they were less likely to engage in sexual intercourse than age-matched peers and tended to initiate intercourse at an older age, 25% began having intercourse aged less than 15 years [20]. The same survey found that teens with CF were not more likely than healthy peers to have sex without a condom, have multiple partners, or not use contraception [20]. The self-reported mean age of first sexual intercourse in Australian young adult males with CF is 17.9 years [60]. Unfortunately, in chronic illness (including CF), sexual and reproductive needs are often ignored by physicians [61]. Knowledge of reproductive health is often poor in adolescents with CF, and physicians may be reluctant or too embarrassed to initiate conversations about sexual health. Reproductive health includes advice about contraception, pubertal delay, sexuality, avoiding sexually transmitted disease, genetic counseling, and treating low fertility. It is imperative that these issues are discussed with adolescents with CF, regardless of any personal beliefs of the physician. A survey of Australian men with CF found that 66% reported wanting more information about reproductive health and wanted this information to come from the CF clinic [60]. In general, patients do not feel comfortable initiating these discussions, would welcome a physician-initiated discussion and feel relieved when being given “permission” to talk about reproductive health. 

Nearly all (98%) of males with CF have azoospermia secondary to bilateral absence of the vas deferens, but a survey of adolescent males with CF has shown that only 60% of adolescents were aware of male infertility [59, 60]. Semen analysis can confirm (and rarely refute) azoospermia, but most adolescents are not aware of this procedure [61]. Young men with CF should be offered semen analysis to establish their fertility status [60]. Regardless of fertility, use of condoms is still required to prevent sexually transmitted diseases. Adolescents must be made aware of this as up to 30% of adolescent males with CF will not use condoms, as they believe they are incapable of causing pregnancy [61]. Another reproductive concern for adolescent males with CF is the small volume of ejaculate; although most had figured this out for themselves, it caused much anxiety and very few had been told that this is normal by a health care professional [60]. Importantly, in the survey conducted by Sawyer et al. [60], 20% of adolescent men and young adult men with CF-confused infertility with impotence, and some were very distressed by this. Discussions about sexuality and reproductive health must occur regularly, with clear and reassuring explanations, no ambiguity and simple language. Various surveys of adults with CF clearly indicate that patients desire information about azoospermia, sperm analysis, and assisted reproduction, and, importantly, most feel that it is a health professional who should initiate discussion [59, 60, 62, 63]. The appropriate age at which to begin these discussions must be decided on an individual basis, but patients indicate that appropriate ages are between the ages of 12 and 16 years [59, 60, 63]. Importantly, some adult men with CF have reported that had they been informed about infertility in adolescence, they would have addressed reproductive options earlier [64]. A useful suggestion by Lyon and Bilton [62] was to include a discussion of fertility and reproduction as part of the “annual review” and transition checklists. Parents should be invited to join discussions about fertility if the adolescent consents. Health professionals should not rely on parents to initiate these conversations with their teenagers, as in the survey conducted by Sawyer et al. [60] only 50% of parents knew that males with CF are usually infertile, and only 30% of parents felt confident that they could discuss this with their teenager even if they were armed with the correct knowledge. 

Contrary to the opinion of many patients, girls with CF may become pregnant—a myth I personally have had to dispel on many occasions. Appropriate contraception must be discussed with all females of reproductive age, and enquiry about sexual activity is part of the routine adolescent H.E.A.R.D.D.S.S. screen. When choosing an appropriate method of contraception there are several practical issues to consider, including poor absorption of the oral contraceptive pill (OCP) with steatorrhoea, interactions with antibiotics (which may affect metabolism, and hence effectiveness of the OCP) and avoiding the OCP in CF hepatic disease. The risk of venous thromboembolism and thrombosis related to implantable venous devices (port-a-cath, infusaports) must be considered, especially when transplant and/or prolonged immobility is likely [64–67]. Pulmonary hypertension is an absolute contraindication to prescription of the OCP. Alternatives to the OCP include injectable progesterone (Depo-Provera); however, given concerns about osteoporosis with long-term use it is best avoided in CF [64]. This is not a concern with progesterone-only implants (Implanon), and this is a suitable, low maintenance option that is not reliable on oral absorption or compliance. Regardless of the method of contraception chosen, condoms must still be used to protect against sexually transmitted diseases. Referral to an adolescent gynaecologist or reproductive specialist may be useful for full discussion of the most appropriate contraceptive methods, and adolescent females may be interested in learning about fertility if they are considering pregnancy in the future. 

As the life expectancy for CF has increased, many people with CF can and do choose to become parents [62]. Genetic counselling must be offered to all adolescents in a sensitive manner, with an explanation of autosomal recessive inheritance of CF using simple diagrams as an aid. It is important not to underestimate the guilt an adolescent may feel when learning that any of their children would be carriers, and that carrier status of potential partners must be considered. Sensitive discussion about the likelihood of the parent's death when children are young must be addressed; this is a very real fear often expressed by people with CF [54, 68–70]. If azoospermic males with CF wish to become parents, they can consider adoption, sperm donation or assisted fertilisation [62]. Sperm can be collected by microsurgical epididymal sperm aspiration (MESA) and then injected into an ovum with intracytoplasmic sperm injection (ICSI) [62], with pregnancy rates of 65.5% and live birth rates between 15 and 55% reported [62, 71, 72]. Females with CF may be subfertile due to thick cervical mucous and poor nutrition preventing ovulation [73, 74], but many women with CF do become pregnant. Careful attention to planning pregnancy is required to avoid irreversible loss of pulmonary function in the mother and premature birth in the infant, avoid teratogenic drugs, and optimise both nutrition and glycaemic control.

## 9. Transition

Transition has been defined as “the purposeful, planned movement of adolescents and young adults with chronic physical and medical conditions from child-centred care to adult-oriented health care systems” [75]. Unfortunately, the transition process does not always run smoothly and was reported as a “disastrous” process by most parents in an Australian CF survey [15]. Although adolescent patients may wish for early transition and find attending a paediatric centre embarrassing or annoying [52], most find transition an upsetting process due to loss of familiarity. It is well recognised that transition can be an anxious time that some adolescents find extremely difficult, and even more problematic when lung transplantation is imminent [76]. Transition may occur when nonadherence becomes a problem and although paediatricians may transition patients from sheer frustration, the adolescent may interpret this as rejection and punishment [51]. Initiating the transition process may be difficult when physicians are reluctant to “let go,” especially when they have cared for an adolescent since infancy, or when they do not trust the ability of the young person to provide their own medical care in an independent manner [51]. 

The key to a successful transition in CF is having a well-established transition program, and most centres have developed their own guidelines. Providing the adolescent with written material is very useful, and “transition checklists” are common resources. Parts of a transition program that have been reported to be particularly useful include tours of the adult hospital and orientation with a trusted member of the paediatric team, joint clinic sessions between paediatric and adult teams, a “familiar face” at adult clinics, and an information package about the new adult hospital [77]. Presenting the transition process as a nonthreatening, routine part of medical care and reinforcing the fact that the adolescent is not being rejected or “sent away” is paramount. There should not be an expectation that transition must be achieved by a certain age or life event (e.g., completion of secondary school), as transition is an individual process that must be tailored and not rushed. A rough goal is that once the routine developmental tasks of adolescence are complete, transition should occur [51]. The adolescent should be independent enough to appropriately manage all aspects of their medical care, have a clear understanding of the structure of the adult clinic, and have visited the site and met team members [51]. Discussion and initiation of the transition process must begin years before it actually occurs, with regular revisiting of the topic and exploration of any anxieties. Discussing transition should be part of the annual review process, and discussion should begin between the ages of 12–15 years, depending on the individual. 

## 10. Lung Transplants

In general, after lung transplantation, the overall median recipient survival is reported to be approximately five years, but survival may be better in CF compared to other pulmonary conditions [78, 79]. Guidelines published by the International Society for Heart and Lung Transplantation can assist in selection of patients who would benefit from and are suitable for lung transplantation [79]. Previously, concerns had been raised that lung transplantation in CF may not actually extend life in children and adolescents, but a thorough review of the evidence by Aurora et al. [80] concluded the contrary if the procedure is timed correctly. Lung transplant may be considered in adolescents with CF who have end-stage pulmonary disease that continues to decline despite maximal therapy with a two-year life expectancy of less than 50% (FEV1 < 30% predicted or PaO_2_ < 55 mmHg or PaCO_2_ > 50 mmHg)[81–84]. Other indications for referral are poor quality of life, frequent infective exacerbations requiring admission, haemoptysis, pneumothorax, oxygen dependence, and pulmonary hypertension [80].

Early referral to a transplant team is useful, as prolonged discussion with the patient and their family is essential to ensure that they have all available information to assist in decision making. Workup for transplant can be a lengthy procedure, and deaths occur while patients wait [85]. Treatment must be optimized, and the transplant team needs to establish a relationship with the adolescent. It is important that adolescents are made aware that a transplant does not change pancreatic insufficiency, there are considerable risks, and they will need to take life-long antirejection medications afterwards. Many adolescents with CF report distress and guilt that for them to live someone else must die, and this must be discussed in a sensitive manner [86]. If they are not known to a psychiatric team, referral is useful so that appropriate support and preparation can occur during the workup. Referral for transplant may present an opportunity to start the transition process although it is advisable to continue seeing the adolescent at both a paediatric and adult hospital when the referral is initially made so that they do not feel abandoned. Referral for transplant can be extremely traumatic, especially as this may be the first time an adolescent has considered their mortality [80]. Even if there is uncertainty about suitability for referral, an appointment with the transplant team can be useful to address concerns that the patient and family may have, and to get a second opinion in regards to suitability.

## 11. End of Life Care

Sadly, CF is still a terminal condition. Adolescents often become acutely aware of this fact and can process and react to this information in many ways. The uncertainty of life expectancy is a major issue for adolescents with CF [15]. The seriousness and ultimate fatality of CF is often emphasised in the media, and this has been shown to be distressing to both patients and their families [15]. The death of a sibling or friend with CF understandably brings his or her own mortality into question. It is not uncommon for adolescents with CF to wonder “My friends seem to be dying, who is next?”

Although life expectancy in CF has increased, deaths still occur in adolescence [1]. The death of a teenager is an extremely distressing time for parents, siblings, extended family, other families who have a child with CF, and health professionals. Literature examining end of life care specifically in CF is rather limited; however, one approach is to combine preventative, therapeutic, and palliative care as the end of life approaches [41]. Although there are effective treatments of distressing symptoms (such as opiates for dyspnoea and chest pain), when a CF patient enters terminal respiratory failure, physicians, allied health staff, family members, and patients themselves may be reluctant to consider care completely palliative [41]. Reasons for this include uncertainty about life expectancy, unwillingness to engage with a new “team,” denial, hope and comfort obtained from continuing a familiar routine [41]. It can be extremely difficult to accurately predict how long a patient will survive when they have acute-on-chronic respiratory failure, hence the unwillingness to discontinue antibiotics and noninvasive ventilation in case of recovery. Even if antibiotics and chest physiotherapy are unlikely to improve prognosis, they may still provide important physical and psychological comfort measures for the patient. Care of a dying child or adolescent is an incredibly difficult situation, as the medical team is responsible for providing not only for the comfort of the patient but of their family. Adolescence is a unique time where the patient may be capable of making all management decisions, and their wishes must be taken into account and respected. Family meetings, support for staff, involving a palliative care team, and including the adolescent in decision making can be useful at this most difficult time. 

## 12. Conclusions

Providing all encompassing health care to a healthy adolescent is challenging, let alone an adolescent with a chronic illness. CF can greatly complicate normal adolescent development and offers numerous additional challenges. Adolescents with CF need to have physicians who are dedicated to addressing and assisting in all areas of health, including medical problems, mental health, reproductive health, and preparation for independent adult life. The unique challenges of adolescence must be accounted for in managing adolescents with CF effectively, and patients value a physician initiating discussion of adolescent issues. Although it can be challenging at times, it is a privilege to be part of an adolescent's journey to adulthood. 
